# Information Warfare-Worthy Jamming Attack Detection Mechanism for Wireless Sensor Networks Using a Fuzzy Inference System

**DOI:** 10.3390/s100403444

**Published:** 2010-04-08

**Authors:** Sudip Misra, Ranjit Singh, S. V. Rohith Mohan

**Affiliations:** 1 School of Information Technology, Indian Institute of Technology, Kharagpur-721302, WB, India; E-Mail: cols@sit.iitkgp.ernet.in; 2 Department of Mathematics, Indian Institute of Technology, Kharagpur-721302, WB, India; E-Mail: svrmohan@iitkgp.ac.in

**Keywords:** wireless sensor networks, jamming detection, fuzzy inference system, information warfare

## Abstract

The proposed mechanism for jamming attack detection for wireless sensor networks is novel in three respects: firstly, it upgrades the jammer to include versatile military jammers; secondly, it graduates from the existing node-centric detection system to the network-centric system making it robust and economical at the nodes, and thirdly, it tackles the problem through fuzzy inference system, as the decision regarding intensity of jamming is seldom crisp. The system with its high robustness, ability to grade nodes with jamming indices, and its true-detection rate as high as 99.8%, is worthy of consideration for information warfare defense purposes.

## 1. Introduction

A large number of wireless sensor networks (WSN)-based applications typically employ numerous inexpensive tiny nodes with on-board sensor(s). They have very limited memory space, energy, and computational power and are interconnected with simplex radio where each node itself acts as a router. The communication is generally limited between a node (source) and the cluster-head/base-station (sink). Since the nodes operate at very low radio power, typically, the transmitted power being a few milli-watts, and the communication range being limited to tens of meters, they are extremely vulnerable to jamming attacks at the physical and data link layers. However, this extreme vulnerability of the WSN to jamming is only one side of the coin. The other side is that, because it is so vulnerable, it is malleable too, and can take the imprint of the jammed area on itself, owing to its high density, spread, and synergy. WSN are, therefore, very suitable for hunting jammers, *i.e.*, detecting, localizing and tracking the jammers for the latter’s eventual liquidation in an information war, which otherwise, is a very costly and difficult task.

### 1.1. Motivation

The authors have chequered experiences of more than twenty-eight years as information warriors, teachers, and researchers and find that detecting the jamming conditions and using it to localize the jammer is a complex and costly affair with the existing technology. WSN have vast military applications for tactical battle field surveillance. However, the same cannot be done effectively because of its high vulnerability to jamming. The authors are motivated to convert this weakness of the WSN into its unique strength of detecting and localizing the jammer. We, therefore, take on the problem of jamming detection in this paper, as our first effort, towards exploiting the potential of WSN in hunting for the jammer in an information war.

There is a special motivation to the approach to the problem too. The existing methods [1–6] of detecting the jamming conditions are node-centric, where the complete data collection, processing and decision making is done by individual nodes to arrive at a crisp decision of ‘jammed’ or ‘not jammed’. This approach is not suitable in a hostile environment because, like wounded soldiers and damaged equipment who/which are graded according to the severity of the casualty/damage, the nodes too must be graded with different jamming indices as per the severity of jamming affecting them. This would make the employment and deployment of the WSN nodes, like any other battle resource, economic and flexible to suit different stages of the information warfare. The other serious drawback of the existing approaches is that the complete processing and decision making is done at the node level. This is not practicable as the WSN nodes are resource-starved and that because nodes may not be able to communicate with others during jamming. Therefore, the authors are motivated to change the approach from decision-making being node-centric to base-station-centric and from the decision being crisp-centric to fuzzy-centric.

### 1.2. Paper Layout

We begin with the discussion of various types of existing jamming attack models in Section 2, such as those from Xu [1], Muraleedharan [2], and Cakiroglu [3]. We then discuss the military classification of jammers, and finally, select our own models relevant to our study from the plethora of existing ones. Section 3 is the study of different possible metrics used for jamming detection and selection of some of them for our work. In Section 4, we analyze the effectiveness and suitability of the existing jamming detection methods, as relevant to the WSN in an information war environment. Section 5 is the description of our proposed mechanism for detecting jamming as well as the type of jammer, followed by the description of the simulation set-up involving NS2, MATLAB, and Simulink simulators in Section 6. In Section 7, we present the performance evaluation of our model and compare it to the other existing ones. We then conclude and outline the future work with Section 8.

### 1.3. Contributions

Our work has a different approach to the problem of detecting jamming, facilitating other jamming related works such as jammed area mapping, jammer localization and tracking and consequent decision making for different anti-jamming actions for various tactical operations. The major contributions are listed as follows:

- *Contour mapping*, based on different lower cut-off values of jamming indices of nodes of the WSN (akin to altitude contours on a geographical relief map) is made possible. This is a better alternative to the present trend of plotting the jammed area, as recommended by different authors such as Wood *et al.* [7], Nowak *et al.* [8], Hellerstein *et al.* [9], and others, because instead of dividing the geographical extent of the WSN into jammed and non-jammed areas, it provides a jamming gradient to the whole area.
- *Flexibility in extent of jammed area mapping* is possible which would give more working space to the battle field commander, e.g., in a defensive battle, during the *pre-contact-with-enemy* stage, when the density and health of the WSN is the best, the jammed area may be bounded by a contour of jamming index of 75% (say) and the same may expand to 50% and 25% during the *contact-with-enemy* stage and *counter-attack* stage, as the battle progresses and the density and health of the WSN goes on depleting.
- *Holistic decision* regarding the jammed condition of a node, based on the node parameters and its neighborhood conditions is taken at the base station, and not at the node level, as done in the existing methods. This not only improves the quality of the decision and survivality of the decision making process, but also takes off the extra burden of taking such decisions from the already resource-starved nodes under siege of a jammer.

## 2. Jamming Attack Models

### 2.1. Military Models for Electronic Warfare

Military jammers have no constraints of energy (power supply) or radiated frequency (RF) power and liberally use their resources with the philosophy of crushing the pea-nut (target network) with the sledge hammer (brute RF power). However, they do exercise restraint and use less RF power only to evade detection. There are three basic jamming attack models used in electronic warfare by the military:

- *Spot Jammer* is a jammer which knows the exact radio frequency of the target network, and attacks the network on that frequency (spot frequency) only. It requires less power to jam the network, and is the most efficient and effective jammer. However, it suffers from the disadvantage that the target network can change the frequency (channel surfing/frequency hopping) to evade jamming.
- *Sweep Jammer is* a jammer which does not know the target frequency, and therefore sweeps across the probable spectrum either periodically or aperiodically, thus jamming the affected networks temporarily. They are less efficient and effective than the spot jammer, but can attack several networks and impose restrictions on freedom of frequency-hopping by the target network.
- *Barrage Jammers* cover a large bandwidth of the radio spectrum at a time, leaving very little scope for the target network to evade jamming. Also, they can jam a number of networks simultaneously. Barrage jammers require high RF power to maintain the required power spectral density of jamming.

### 2.2. Jamming Attack Models from Academia

#### 2.2.1. Models of Xu *et al.* [1]

Xu *et al.* [1] have suggested four types of models, described below:

- *Constant Jammer* is not aware of the existing protocols of the network (bit-rate, packet-size etc.) and, therefore keeps transmitting bits constantly over a period of time without following any protocol. They are not energy efficient.
- *Deceptive Jammer* is aware of the target network’s protocol and jams the network by transmitting legitimate packets constantly over a period at a high rate to keep the carrier captured. It is highly effective but is as energy inefficient as the constant jammer.
- *Random Jammer* functions either like a constant jammer or a deceptive jammer but does so randomly. It is less effective than the jammer whom it imitates (constant or deceptive) but is more energy efficient than it.
- *Reactive Jammer* also knows the communication protocols of the target network. It keeps listening to the network passively, and attacks the network at its chosen time in a manner as if it is part of the network, following its protocols. It is most effective but not very energy-efficient as it spends considerable amount of energy in constantly listening to the network.

#### 2.2.2. Models of Law *et al.* [11]

The S-MAC protocol has these time segments: synchronization, listening, control, data, and sleep. Law *et al.* [11] have suggested four types of energy-efficient jammers for attacking a network following the S-MAC protocol:

- *Periodic Listening Interval Jammer* attacks when the nodes are in listening period and sleeps at all other times.
- *Periodic Control Interval Jammer* attacks when the nodes are in the control period and sleeps during rest of the time.
- *Periodic Data Packet Jammer* listens to the channel during the control interval and attacks the data segment.
- *Periodic Cluster Jammer* is meant for attacking networks following encrypted packets. It uses k-means clustering algorithm to separate clusters of the network and statistical estimations to determine the timing of the data segment, and then attacks the same accordingly.

#### 2.2.3. Models of Wood *et al.* [12]

Wood *et al.* [12] have also suggested four jamming attack models, described below:

- *Interrupt Jammer* is a variation of *Reactive Jammer* in the sense that instead of listening to the channel constantly, it gets activated by means of a hardware interrupt when a preamble and start of frame delimiter (SFD) are detected from a received frame.
- *Activity Jammer* is yet another variation of *Interrupt Jammer* (in fact, that of a *Reactive Jammer*) meant for encrypted packets where detection of the SFD is other-wise not possible.
- *Scan Jammer* is similar to the *Sweep Jammer*. Instead of detecting a packet in a single channel, it searches out all possible channels for a packet during a defined period of time, and having succeeded, it then attacks the channel.
- *Pulse Jammer is akin* to the *Constant Jammer* in the sense that it sends small packets constantly to jam a channel.

#### 2.2.4. Models of Muraleedharan *et al.* [2]

Muraleedharan *et al.* [2] have described four models: *Single-Tone Jammer, Multi-Tone Jammer, Pulsed-Noise Jammer*, and *Electronic Intelligence (ELINT)*. The *Single-Tone Jammer* attacks one channel at a time (akin to *Spot Jammer*), the *Multi-Tone Jammer* can attack some or all the channels of a multi-channel receiver, while the *Pulsed-Noise Jammer* is a wide band jammer, sending pulsed jamming signals by turning on and off periodically at a slow or fast rate. *ELINT*, as they describe, is typically a passive system that tries to break down or analyze radar or communication TCF signals, and thus, strictly speaking, is not a jamming attack model.

### 2.3. Analysis of the Existing Models

Study of the aforesaid models reveals that while the military models are focused towards attacking the network at the physical layer (thus, attacking all the other upper six layers like shaking the foundation of a tall building and affecting all the upper storeys consequently) with RF power being their main weapon (since there are hardly any energy and RF power constraints), the academic models are focused towards attacking the data-link layer with RF power levels at par with the existing average transmitted power of a WSN node. It also brings to the forefront the difference in approach to identification of the attacker. The academics seems to believe that the attacker (jammer) is a small time player with limited resources, who is either an intruder or one of our own compromised nodes, fully or partially knowing the protocols, and attacking the network with stealth as its main weapon from a location well within the geographical extent of the WSN. The military believes that it is neither worth the effort to learn the WSN protocols nor essential to move into the WSN geographical area for jamming, since it is so easy to jam the nodes with brute RF power from a far-off safe distance, especially when the RF frequency is known. Therefore, there is a need to balance the two approaches in modeling the jamming attack to make our counter-jamming efforts, like jamming detection and jammer localization, suitable for information warfare.

### 2.4. Description of the Proposed Jamming Attack Models

Based on the foregoing discussion and recognizing both, the jammer’s transmitted RF power and the knowledge/ignorance of the target network’s communication protocols to the jammer, we propose the following jamming attack models, which are in fact, the derivatives of the models proposed by Xu *et al.* [1], redefined to suit the information warfare requirements:

1. *Constant Jammer with Normal Power (CON)* is a constant jammer with transmitted RF power comparable with the average RF transmitted power of the target WSN.
2. *Constant Jammer with High Power (COH)* is a constant jammer with high transmitted RF power.
3. *Deceptive Jammer with Normal Power (DECN)* is a deceptive jammer with transmitted RF power comparable with the average RF transmitted power of the target WSN.
4. *Deceptive Jammer with High Power (DECH)* is a deceptive jammer with high transmitted RF power.
5. *Random Jammer Imitating CON*, *(RACN)*.
6. *Random Jammer Imitating COH*, *(RACH)*.
7. *Random Jammer Imitating DECN*, *(RADECN)*.
8. *Random Jammer Imitating DECH*, *(RADECH)*.
9. *Reactive Jammer with Normal Power (REN)* is a reactive jammer with transmitted RF power comparable with the average RF transmitted power of the target WSN.
10. *Reactive Jammer with High Power (REH)* is a reactive jammer with high transmitted RF power.

## 3. Metrics for Jamming Attack Detection

Xu *et al.* [1] define a jammer *‘to be an entity who is purposefully trying to interfere with the physical transmission and reception of wireless communications’*. This can be achieved by the jammer by attacking at the physical layer or at the data-link layer. At the physical layer, the jammer can only jam the receiver by transmitting at high power at the network frequency and lowering the signal-to-noise ratio below the receiver’s threshold; however, it cannot prevent the transmitter from transmitting, and hence it cannot jam the transmitter. At the data link layer, it can jam the receiver by corrupting legitimate packets through protocol violations, and can also jam the transmitter by preventing it to transmit by capturing the carrier through continuous transmission (another form of protocol violation). With this *modus operandi* of the jammer at the background, we examine the suitability of various metrics, as suggested by different scholars, for detecting jamming attack on a WSN.

### 3.1. Carrier Sensing Time (CST)

In Media Access Control (MAC) protocols, such as Carrier Sense Multiple Access (CSMA), each node keeps sensing the time for the carrier to be free so that it can then send its own packets. The average, time period for which the node has to wait for the carrier (channel) to become free and available to it is called the Carrier Sensing Time (CST). It is calculated as the mean of the time duration elapsed between the instant a node is ready to send its packet and the instant at which the carrier is found free by it for sending its packet. The nodes fix a threshold value of the CST, which if exceeded, allows it to infer that there is a jamming attack aimed at capturing the carrier. The threshold can either be fixed, as in case of 1.1.1 MAC, or taken as the minimum value over a given time period, as done in case of BMAC. This metric can be applied to only those networks using a MAC protocol based on carrier sensing. Also, this metric is incapable of indicating a physical layer power attack. It also suffers from the problem of fixing thresholds, which is an imprecise process and is computationally taxing on the scarce resources of the WSN node. We therefore, do not find it suitable for our system.

### 3.2. Packet Send Ratio (PSR)

Xu *et al.* [1] define PSR of a node as the ratio of the number of packets actually sent by the node during a given time period to the number of packets intended to be sent by the node during that given period. ‘The number of packets intended to be sent during a given time period’ is found by calculating the time of the channel’s availability to the node during the given period, much in the same way as in the case of CST, and then by multiplying this available time with the packet transmission rate. Finally, the PSR is calculated as defined above. The PSR-calculation is cumbersome and accordingly, we do not find it suitable for our system either.

### 3.3. Packet Delivery Ratio (PDR)

Both, Xu *et al.* [1] and Cakiroglu *et al.* [3] define PDR as the ratio of the number of packets successfully sent out by the node (*i.e.*, the number of packets for which the node has got the acknowledgement from the destination) to the total number of packets sent out by the node. Xu *et al.* [1], however, define two types of PDR: firstly, one to be measured by the transmitter (source), and secondly, one to be measured at the receiver (sink). We, while talking of the PDR, mean only the first one, *i.e.*, the one measured at the transmitter-end, and shall discuss the second type, *i.e.*, the one to be measured at the receiver-end, separately. The PDR is calculated by keeping counts of the acknowledgements of the successfully delivered packets and the total number of packets sent by the node and then by finding their ratio as a percentage. PDR is a very good metric which is capable of being measured accurately by the node without much of computational overhead, and can indicate the presence of all types of jamming attacks at the physical or data-link/MAC layer. However, the necessary condition is that the network must follow a protocol, like TCP, where the system of acknowledgement of packets exists. We feel that a resource- starved network, like the WSN, cannot afford the luxury of acknowledgements, and hence reject it from our choice.

### 3.4. Bad Packet Ratio (BPR)

BPR is same as that PDR which is to be measured at the receiver-end, as suggested by Xu *et al.* [1]. However, Cakiroglu *et al.* [3] call it BPR and define BPR as the ratio of the number of bad packets received by a node (*i.e.*, the number of received packets which have not passed the Cyclic Redundancy Check (CRC) carried out by the node) to the total number of packets received by the node over a given period of time. We find BPR to be a very effective metric which can indicate all types of jamming, is easily calculable, and is fit for WSN where the system of acknowledgements is not required. The CRC is a normal procedure which nodes have to do under most of the existing protocols to check whether a received packet is correct or erroneous. If the packet is correct (good packet), it is received or queued for further transmission, and if the packet is erroneous (bad packet), it is dropped and their count is maintained. Therefore, both values, the number of bad packets and the number of total received packets, are readily available for computing the BPR without imposing any significant burden on the system. Also, there is no sampling or fixing of thresholds involved here. We find this metric suitable for our system.

### 3.5. Standard Deviation in Received Signal Strength (SDRSS)

Reese *et al.* [4] have suggested a system where the node samples its received legitimate signal, called the clean signal, over a period of time and finds its standard deviation (σ) during the period. It then samples the abnormal signal, called the jammed signal, and finds its mean deviation (đ) from the clean signal over the same period of time. The calculation of σ and đ are done as per formulae [4]. If đ ≤ σ, then there is no jamming; else, there is jamming. Although we will discuss SDRSS subsequently under the method suggested by Reese *et al.* [4], we do not find it suitable for the WSN due to: (1) it cannot work if the jammer is transmitting at a power level equal to the normal transmitted power level of the nodes, as it would do during many types of jamming attacks, like deceptive jamming, as discussed above, (2) it involves sampling at the node level, and (3) it is computationally taxing for a WSN node.

### 3.6. Bit Error Rate (BER)

Strasser *et al.* [5] have recommended the use of BER in combination with the received signal strength (RSS), as it is not only a very effective metric for detecting jamming attack, but is also capable of identifying the reactive jamming attack, which otherwise is very difficult to identify. The BER is calculated as the ratio of the number of corrupted bits to the number of total bits received by a node during a transmission session. We concur with the authors as far as the effectiveness of this metric is concerned, but find the calculation of the BER heavily taxing for a WSN node, especially in a networking environment where the node will have to keep track of the BER of all radio links with its one-hop neighbors. Calculation and updating of BER, even at the base station level, is not feasible because it involves collection of voluminous data regarding every bit of a valid and invalid packet from the nodes leading to over-taxing of the WSN.

### 3.7. Received Signal Strength (RSS)

The received signal strength is defined as the power content of the radio signal received at the receiver. It is a measurable quantity and can either be measured by the RF power meter of the node or can be calculated using formulae as per the selected propagation model. The RSS by itself is not a logical metric to indicate jamming. However, when used in combination with metrics like the received jammer power (or noise power) or BER, it forms an effective combination to detect jamming.

### 3.8. Signal-to-Noise Ratio (SNR) or Signal-to Jammer Power Ratio (SJR)

Although there is a subtle difference between SNR and SJR, we have considered these to be the same because, in our model jammer is the predominant noise source, and have used these terms interchangeably. SNR is calculated as the ratio of the received signal power at a node to the received noise power (or jammer power) at the node. It is almost an effective metric to identify a jamming attack at the physical layer as there can be no jamming at the physical layer without the SNR dropping low. However, some other metrics like PDR, BPR, or BER which can identify a data-link/MAC layer attack should be used with SNR for making it almost full- proof to detect jamming.

### 3.9. Energy Consumption Amount (ECA)

Cakiroglu *et al.* [3] define ECA as the *approximated energy amount consumed in a specified time for a sensor network.* It can be calculated by measuring the drop in the battery (power-supply) voltage (*v*) of the node and multiplying its squared value with the time duration and then by dividing the result with the average electrical load (resistance) of the node. The authors argue that certain jammers force sensor nodes to remain in BACKOFF period even if they should have switched to IDLE mode, causing them to consume more energy than the normal. They suggest that this consequence can be used to distinguish the normal and jamming scenarios from each other. This metric has two pit falls: firstly, the sampling of the threshold energy consumptions of the node by itself under different traffic-load conditions is a tall order, and secondly, there may not be any perceptible energy consumption differential when the jammer is attacking in a way which does not involve the carrier capture, or when the jammer is resorting to simple power attack.

### 3.10. Selected Metrics for the Proposed System—SNR and BPR

We select SNR and BPR as the jamming attack metrics for our system. However, we prefer to call the BPR as Packets Dropped per Terminal (PDPT) because our PDPT is the average BPR of a node during a simulation cycle. The reasons for this choice have been discussed above, and the same are summarized as follows:

- The received radio power at a node is easily measurable as nodes are/can be provided with RF power meter.
- In our system, the node simply keeps the base station informed about the received radio power, at a time interval as decided by the base station. The base station calculates the jammer (noise) power by subtracting the average legitimate signal power of the node from the current power. The ratio of the two powers is then calculated by the base to get the SNR. Thus there are no over-heads involved at the node level.
- The node keeps the base station informed about the number of good packets and total packets received by it during a time interval, as decided by the base station, in a normal routine way. The base station calculates the BPR (or, PDPT) for each node. Thus, the nodes are not burdened additionally.
- The combination of SNR and BPR (or, PDPT) is capable of detecting any form of jamming attack, as discussed in the previous sections.

## 4. Existing Jamming Attack Detection Methods and Their Analysis

Several scholars have suggested different mechanisms to detect jamming attacks. All of these suggested mechanisms are to be implemented at the individual node level to crisply conclude whether the node is jammed or not. Their technique is either based on threshold values of some of the metrics, as discussed before, or to use digital signal processing techniques to differentiate between a legitimate signal and an illegitimate (jamming) signal and thus conclude about the presence or absence of the jammer. Few of the methods use comparison of the node conditions with those of its neighbors to fine-tune their findings. We discuss the existing solutions, as proposed by different scholars in the following sub-sections.

### 4.1. Studies by Xu *et al.* [1]

Xu *et al.* [1] carried out intense study of the jamming attack detection mechanism with experiments using the MICA2 Mote platform. Firstly, they collected data about various percentages of the PSR and PDR (measured at the transmitter end) for constant, deceptive, random, and reactive jammers for BMAC and 1.1.1 MAC protocols for varying distances between the transmitting-node and the jammer. They considered additional jammer parameters like on-off periods for the random jammer and different packet sizes for the reactive jammer. The results show that although the PSR and the PDR vary for different jammers under different conditions, it is difficult to conclude about jamming and its type by these parameters alone. They then studied the levels of carrier sensing time, energy consumption, and the received signal strength as well as the received signal spectrum under normal and jamming conditions for two application layer protocols: Constant Bit Rate (CBR) and Maximum Traffic and tried to identify the jammer type through spectral discrimination using the Higher Order Crossing (HOC) method [14]. They conclude through these experiments that it is not always possible to use simple statistics, such as average signal strength, energy, or carrier sensing time to discriminate jamming condition from the normal traffic, because it is difficult to devise thresholds. They also conclude that the HOC method can distinguish the constant and deceptive jamming from the normal traffic, but cannot distinguish the random and reactive jamming from the normal traffic. Finally, they conclude that if PDR is used with consistency checks like, checking own PDR and signal strength and comparing the same with those of the neighbors, and/or ascertaining own distances from the neighbors, then the combination can very effectively detect and discriminate various forms of jamming.

The study is rigorous and the suggested methodology is sound. Its limitations are: (1) the complete process has to be done by the WSN node which is taxing, and (2) that the node may not be able to communicate with its neighbors during jamming to get the required statistics for comparison, as required in the method.

### 4.2. Method Suggested by Rajani *et al.* [2]

Rajani *et al.* use ‘*the swarm intelligence and ant system’* wherein they create an agent (ant) which proactively uses the WSN node’s information (key performance parameters), as it traverses a route from node to node, to predict or anticipate jamming, and accordingly, changes the route to avoid jamming. They suggest a decision threshold, called probability of selecting a link between nodes *i* and *j*, called *P_ij_*_,_ to be calculated at node *i*. They describe that if the calculated *P_ij_* is within the acceptable limits then the agent selects the link for its travel, else, it rejects it and selects that link whose *P_ij_* is within the acceptable limits. *P_ij_*_,_ as suggested by them, is to be calculated using complicated formulae.

Although, the approach to the problem is novel, it is obvious that it is not workable for detecting a jamming attack, especially in an information warfare environment, because: (1) some of the data, e.g., packet delivery ratio and the packet loss on a link will not be readily available normally, and if they are to be kept readily available, they will be at the cost of memory space of the node, (2) some of the data, like BER,are extremely complicated to be ascertained (as already mentioned and to be described in detail later) and involve communicating with other nodes, which may not be possible under jamming, (3) it is computation-intensive and taxes the resource-starved WSN node, (4) it involves of fixing threshold of the decision parameter for each node under different conditions, which is fraught with pit-falls, as discussed earlier, and (5) it is based on evolutionary algorithms whose complexities in terms of time and space is difficult to ascertain; but are important to be minimized for any resource-constrained network, like the WSN.

### 4.3. Method Suggested by Cakiroglu *et al*. [3]

Cakiroglu *et al.* [3] have proposed two algorithms for detecting a jamming attack. The first algorithm is based on threshold values of three detection parameters: Bad Packet Ratio (BPR), Packet Delivery Ratio (PDR), and Energy Consumption Amount (ECA). If all three parameters are below the thresholds, or if only the PDR exceeds the threshold, then it is concluded that there is no jamming; otherwise, there is jamming. The second algorithm is an improvement over the first one where the neighboring nodes’ conditions, ascertained through queries to be raised and replies there-to to be received within the threshold time periods, are also taken into account to enhance the jamming detection rate. The results of the simulations are very encouraging, thus establishing the effectiveness of the algorithms. However, the suggested models suffer from fixing of too many thresholds and processing at the node levels, which have their own problems, as discussed earlier. In addition, the PDR, measured at the transmitter-end, as in the instant case, is not suitable for the resource-constrained WSN because it imposes the avoidable burden of acknowledgements.

### 4.4. Method Suggested by Reese *et al.* [4]

Reese *et al.* [4] have proposed a method to differentiate a legitimate received signal, called clean signal, *c(t)* from a signal received from a jammer, called jammed signal, *j(t)* based on the standard deviation of the clean signal, *σ* and the mean difference between the clean and jammed signals, *d̄*, averaged over a period of time *t* from *t = a* to *t = b*, where, *a* and b are chosen constants. They first compute the root mean square value of the clean signal, *C_rms_*_,_ and then use it for other computations.

If *d̄* is greater than σ, they conclude that it is a jamming signal; else, it is a clean signal. As evident, the calculations have to be done by the WSN node over a period of time (from *t = a* to *t = b*), very frequently, almost all the time, to keep differentiating the clean and jammed signals. This is a great disadvantage of this method, if used for jamming detection in the WSN scenario. Also, it cannot discriminate a jamming signal, if the jammer uses the same power in the jamming signal as that in the clean signal.

### 4.5. Method Suggested by Strasser *et al*. [5]

Strasser *et al.* [5] have suggested a very effective method of detecting a reactive jammer (which otherwise is so difficult to be detected) through Received Signal Strength (RSS) and Bit Error Rate (BER) samplings and inferring the presence of the reactive jammer in the event of high BER despite the RSS being normal or better than the normal. The method involves three steps: (1) error sample acquisition, (2) interference detection, and (3) sequential jamming test to infer presence or absence of reactive jamming.

Error sample acquisition is done in two sub-steps: (a) packet reception and RSS recording, *i.e.*, forming the tuple (m, s), where, m is the sampled message packet and s is the corresponding RSS, and (b) identifying the BER.

They have suggested three methods for ascertaining whether a bit is correct or erroneous: (i) by XOR-ing the instant bit, m(i) with the predetermined value of the bit, m’(i) and concluding that the instant bit is faulty if the XOR result is true, (ii) using Error Correcting Codes (ECC), and (iii) through wired node chain (n-tuples) system, in which they generate error sample, e(i) as the result of theXOR of the bit received on the wireless link, wl(i) and the bit received by the same node from the same transmitter, transmitted simultaneously on a wired link, w(i), *i.e.*, e(i) = wl(i) XOR w(i), and take the minimum of the RSSs received on the wireless and the wired links as the corresponding RSS, *i.e.*, s(i) = min [swl(i), sw(i)] and form tuple [e(i), s(i)].

In the second step, interference detection, they confirm the presence of interference if e (i) = 1 (true) and s (i) > S, where S is a predetermined threshold value of the RSS, and confirm absence of interference otherwise.

In the third step, sequential jamming test, they take decision regarding presence or absence of jamming based on the values of three decision parameters: likelihood ratio *η(k)*, targeted probability of false alarm being true *T_FP_*, and targeted probability of false alarm being not true *T_FN_*.

The method has a sound mathematical foundation and is capable of detecting all types of jamming attacks, including reactive jamming, but it cannot discriminate different types of jamming attacks. It also involves of sampling/fixing thresholds and values like those for *p_c_*, *T_FN_*, *TFP*_,_ and *S*, the RSS threshold, which have peculiar problems, as discussed before. Besides, it is taxing on a WSN node in terms of computational and memory-space resources.

### 4.6. Comparison of Existing Methods

A comparative study of the existing jamming detection methods is as given in Table 1.

### 4.7. Conclusions from Study of Existing Methods

Some major deductions from the discussion of various jamming-detection methods and their comparative study are: that none of the methods, except Xu’s method, is capable of discriminating various types of jamming; that they all tax the scarce resources of the nodes; that the decision is taken by the nodes based on only its own parameters and those of its neighbors (if spared by the jammer to get these) without being aware of the global scenario, and that their detection-quality is highly dependent on the ability of the victim nodes to continue communicating with their neighbors or the base station despite adverse jamming conditions. The existing methods are capable of detecting all jammers, including military warfare jammers; yet they are not suitable for information war because they are unable to grade the intensity of jamming experienced by different victim nodes, which is vital for further decision making by the battle field commander (or the base station). These inferences lead us to conclude that the existing methods are vulnerable to different jamming attacks under organized information warfare and, as such, there is a need to devise a method to obviate the vulnerabilities and improve the detection quality with the added ability of quantifying the intensity of jamming at different nodes.

## 5. Proposed Method

### 5.1. Description

We now propose a fuzzy inference system-based jamming detection method which follows a centralized approach, wherein the jamming detection is done by the base station based on the input values of the jamming detection metrics received by it from the respective nodes. There are three inputs required to be sent by the nodes to the base station: 1) the number of total packets received by it during a specified time period, 2) the number of packets dropped by it during the period, and 3) the received signal strength (RSS). The former two metrics are normally sent to the base as part of the network health monitoring traffic at a pre-decided frequency, as part of most of the existing network management protocols. The third metric, RSS has to be additionally sent to the base station in our scheme. This can be preferably sent packaged with the former two parameters, or else, sent independently. The base station computes the ‘power received by the node from the jammer’, if any, by finding the differential between the current RSS and normal RSS values. Thereafter, the base station computes the PDPT and SNR from these values, as discussed before. Then the base station uses the values of PDPT and SNR as inputs to a fuzzy inference system (Mamdani’s Fuzzy Inference System’) to get ‘Jamming Index’ (JI) as output of the system. The JI value varies from 0 to 100, signifying ‘No Jamming’ to ‘Absolute Jamming’ respectively. In this way, the base station is able to grade the intensity of jamming being experienced by each node through the JI parameter, and thus build an overall picture.

The base station, through the overall picture that it has, is now able to do a confirmatory check through neighborhood study of any node to ascertain the correctness of the JI grade allotted to that node, as compared to the JI allotted to its neighbor nodes. This is done in our method through an algorithm called ‘2-Means Clustering of node Neighborhood’. The elegance of the method lies in doing away with the requirement of communicating with the neighbor nodes for neighborhood check. This enhances the survivality of the system during jamming.

Now, depending upon the overall picture and the battle field conditions, the battle field commander (or the base station) can decide the lower cut-off value of JI to conclude that all nodes whose JIs are greater than the lower cut-off value are ‘Jammed’ while the others are ‘Not Jammed’. The further details are described in the sub-sections that follow.

#### 5.1.1. Detection of Jamming Attack on a Node Using Fuzzy Inference System

Definition: Fuzzy Sets and Membership Functions

Jang *et al.* [17] define fuzzy sets and membership functions as below.

If *X* is a collection of objects, called the universe of discourse (uod) denoted generically by *x*, then a fuzzy set *A* in *X* is defined as a set of ordered pairs:

(1) $$A=\left\{\left(x,\mu_{A}(x)\right):x\in X\right\}$$

where *μ_A_* (*x*) is called the membership function (MF) for the fuzzy set *A*. The MF maps each element of *X* to a membership grade (or membership value) between 0 and 1.

In simple terms, fuzzy means one which cannot be quantified crisply, e.g., the set *‘Tall’* defined over the universe of discourse, ‘*Height’* (measurable in cm), may mean different things for different people. Some may consider persons of height 180 cm or more to be tall, while for others a person of height 175 cm may also be tall. Therefore, the set ‘*Tall*’ is a fuzzy set. It must be noted that while the set ‘Tall’ defined over universe of discourse ‘*Height*’ (which may generically be denoted by *h*), is fuzzy, the universe of discourse ‘*Height*’ is a crisp set because its members will assume crisp quantifiable values in cm.

Fuzzy logic is a computational paradigm that provides a mathematical tool for representing and manipulating information in a way that resembles human communication and reasoning process [15]. We define three fuzzy sets each over the two universes of discourse (inputs), SNR and PDPT: LOW, MEDIUM, and HIGH. Four fuzzy sets are defined over the universe of discourse, JI: NO (meaning normal), LOW, MEDIUM, and HIGH. We use Mamdani model [16], where SNR and BPR (or, PDPT) are the crisp inputs to the system and JI is the crisp output obtained from the system after defuzzification using the centroid method.

##### 5.1.1.1. Fuzzification Process

Multiple sets of two crisp inputs, SNR and PDPT, as generated through NS2 simulations (the simulation set up will be described in Section 6) are first mapped into fuzzy membership functions. A trapezoid shape is chosen to define fuzzy membership functions, because of two reasons: firstly, it can be mathematically manipulated to be very close to the most natural function, the Gaussian or Bell function, and secondly, it can be easily manipulated to be an unsymmetrical function (as required in the instant case) where the same cannot be done so easily with the Gaussian or Bell functions.

**Figure 1.**
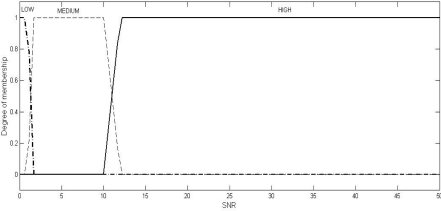
Graphical representation of the trapezoidal function for the input signal-to-noise ratio (SNR).

**Figure 2.**
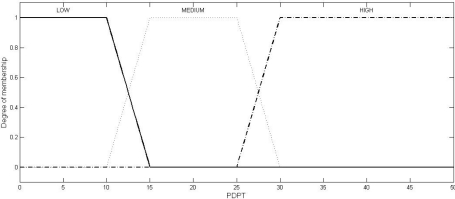
Graphical representation of the trapezoidal function for the input bad packet ratio (BPR) or packets dropped per terminal (PDPT).

**Figure 3.**
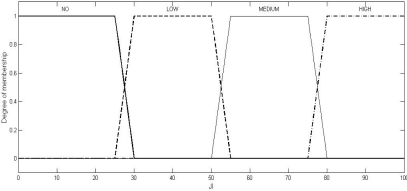
Graphical representation of the trapezoidal function for the output, jamming index (JI).

We define the membership functions below:

(2) $$\mu_{\mathrm{set}}\;(\mathrm{uod})=\{\begin{array}{ll} \frac{\mathrm{uod}-a}{b-a}, & a\leq \mathrm{uod}\leq b\\ 1, & b<\mathrm{uod}<c\\ \frac{d-\mathrm{uod}}{d-c}, & c\leq \mathrm{uod}\leq d\\ 0, & \mathrm{otherwise} \end{array}$$

where the different values of the variables are as given in Table 2. The values of the variables, as shown in Table 2, have been fixed through two stages: firstly, as per the mean of the values obtained from the experts, and secondly, by the correction of these values through a feed-back factor generated by comparing the actual result (the output, JI of the system) and the expected result (the JI value, as expected by the experts). The graphical representations of these trapezoidal functions in respect of SNR, PDPT, and JI are shown in Figures 1, 2, and 3, respectively.

##### 5.1.1.2. Fuzzy Inference

The second step in fuzzy logic processing is fuzzy inference. A rule base, comprising of the range of rules consisting of fuzzy outputs corresponding to SNR and PDPT fuzzy inputs, was formed using the opinion of experts with rich theoretical and practical experience in jamming and counter jamming disciplines of information warfare. The rule base was further refined by vetting the system outputs by the experts. The rule base is given as follows:

1. If SNR is LOW and PDPT is LOW then JI is HIGH.
2. If SNR is LOW and PDPT is MEDIUM then JI is HIGH.
3. If SNR is LOW and PDPT is HIGH then JI is HIGH.
4. If SNR is MEDIUM and PDPT is LOW then JI is LOW.
5. If SNR is MEDIUM and PDPT is MEDIUM then JI is MEDIUM.
6. If SNR is MEDIUM and PDPT is HIGH then JI is HIGH.
7. If SNR is HIGH and PDPT is LOW then JI is NO.
8. If SNR is HIGH and PDPT is MEDIUM then JI is LOW.
9. If SNR is HIGH and PDPT is HIGH then JI is MEDIUM.

**Figure 4.**
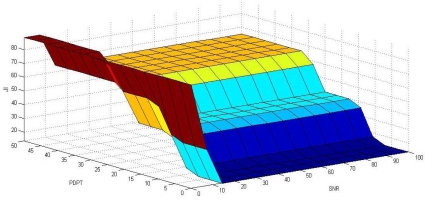
Input-output surface corresponding to the membership values of inputs (SNR, PDPT) and output (JI).

Relations obtained from the rule base are interpreted using the minimum operator ‘and’. The outputs obtained from the rule base are interpreted using maximum operator ‘or’. The overall input-output surface corresponding to the above membership functions, values of variables, and rule base is depicted in Figure 4.

##### 5.1.1.3. Defuzzification

The outputs of the inference mechanism are fuzzy output variables. The fuzzy logic controller must convert its internal fuzzy output variables into crisp values, through the defuzzification process, so that the actual system can use these variables. Defuzzification can be performed in several ways. We choose the Centroid of Area (COA) method [17]. In this method, the centroid of each membership function for each rule is first evaluated. The final output, JI which is equal to COA, is then calculated as the average of the individual centroid weighted by their membership values as follows:

(3) $$\mathrm{JI}=\mathrm{COA}=\frac{\sum_{\mathrm{uod}=a}^{b}\mu_{\mathrm{set}}(\mathrm{uod}).\mathrm{uod}}{\sum_{\mathrm{uod}=a}^{b}\mu_{\mathrm{set}}(\mathrm{uod})}$$

where, *JI* or *COA* is the defuzzification output, *uad* and *μ_set_(uad)* are input variables and their corresponding minimum/maximum values of membership degrees. The complete process of calculating the crisp value of jamming index (JI) from input values of SNR and PDPT for every WSN node is done with MATLAB-7.

#### 5.1.2. Confirmation of Jamming Attack on a Node Through ‘2-Means Clustering’ of Node Neighborhood

After each node has been assigned a crisp jamming index (JI) as per its SNR and PDPT values by the base station through the aforesaid method, the base station now confirms whether a node can be declared jammed or not jammed by looking at the jamming indices of neighboring nodes. This is done by the base station as follows:

1. Depending upon the information war conditions, it decides the lower cut-off value of *JI*, *LC* for declaring all nodes with *JI* ≥ *LC*, as jammed nodes, *i.e.*, jamming detected at these nodes.
2. It makes a list of all jammed nodes, *i.e.*, of nodes having *JI* ≥ *LC* and finds the number, *t* of such nodes.
3. For each of the *t* jammed nodes, it does the following:
  - Identifies and counts the number of one-hop neighbors, *n*.
  - Out of the *n* neighbors, it identifies those neighbors who are in the list of jammed nodes and counts their number, *nj* and names the group of these nodes as *jammed neighbors cluster.*
  - Out of the *n* neighbors, it identifies those neighbors who are not in the list of jammed nodes and counts their number (*n*- *nj*) and names the group of these nodes as *non-jammed neighbors cluster.*
    We thus have a total of *n* nodes divided into 2 clusters in neighborhood of a node under consideration. Therefore, the deciding figure is n/2. If the number of nodes (*nj*) in the *jammed neighbors cluster* is more than n/2 then majority of the neighbors are jammed and hence it is confirmed that the node under consideration is also jammed. If *nj is less than or equal to n/2*, further examination is required for taking any decision. The subsequent steps of the algorithm proceed accordingly.
  - If *nj* > *n/2*, then it confirms that the node is jammed.
  - If *nj* ≤ *n/2*, then it does the following:
    - Finds the mean jamming index of *jammed neighbors cluster*, $\bar{\mathrm{jij}}$ using the formula:
      (4) $$\bar{\mathrm{jij}}=\frac{\sum_{k=1}^{\mathrm{nj}}{\mathrm{ji}}_{k}}{n_{j}}$$
    - Finds the mean jamming index of *non-jammed neighbors cluster*, $\bar{\mathrm{jinj}}$ using the formula:
      (5) $$\bar{\mathrm{jinj}}=\frac{\sum_{k=1}^{n-\mathrm{nj}}{\mathrm{ji}}_{k}}{n-n_{j}}$$
    - Finds centroid X and Y coordinates of *jammed neighbors cluster* using the formula:
      (6) $$\left(\bar{x_{j}}=\frac{\sum_{k=1}^{\mathrm{nj}}{\mathrm{ji}}_{k}.x_{k}}{\sum_{k=1}^{\mathrm{nj}}{\mathrm{ji}}_{k}},\;\;\;\;\;\;\;\;\;\bar{y_{j}}=\frac{\sum_{k=1}^{\mathrm{nj}}{\mathrm{ji}}_{k}.y_{k}}{\sum_{k=1}^{\mathrm{nj}}{\mathrm{ji}}_{k}}\right)$$
    - Finds centroid X and Y coordinates of *non-jammed neighbors cluster* using the formula:
      (7) $$\left(\bar{x_{\mathrm{nj}}}=\frac{\sum_{k=1}^{n-\mathrm{nj}}{\mathrm{ji}}_{k}.x_{k}}{\sum_{k=1}^{n-\mathrm{nj}}{\mathrm{ji}}_{k}},\;\;\;\;\;\;\;\;\;\bar{y_{\mathrm{nj}}}=\frac{\sum_{k=1}^{n-\mathrm{nj}}{\mathrm{ji}}_{k}.y_{k}}{\sum_{k=1}^{n-\mathrm{nj}}{\mathrm{ji}}_{k}}\right)$$
    - Finds the square of the distance, *d_j_* of the node under consideration from the centroid of the *jammed neighbors cluster* using the formula:
      (8) $$d_{j}^{2}={\left(x-\bar{x_{j}}\right)}^{2}+{\left(y-\bar{y_{j}}\right)}^{2}$$
    - Finds the square of the distance, *d_nj_* of the node under consideration from the centroid of the *non*-*jammed neighbors cluster* using the formula:
      (9) $$d_{\mathrm{nj}}^{2}={\left(x-\bar{x_{\mathrm{nj}}}\right)}^{2}+{\left(y-\bar{y_{\mathrm{nj}}}\right)}^{2}$$
    - If:
      $$\left(\bar{\mathrm{jij}}/d_{j}^{2}\right)\geq \left(\bar{\mathrm{jinj}}/d_{\mathrm{nj}}^{2}\right),$$
      then it declares that the node is jammed; otherwise, it declares that the node is not jammed and then deletes its name from the list of jammed nodes.

## 6. Simulation Set-up and Configuration

### 6.1. Simulation Parameters for WSN and Jammers

The details of the input parameters used for the simulation in respect of the WSN and those parameters which are globally applicable to the jammer-simulation are given in Table 3.

### 6.2. Special Simulation Parameters for Different Types of Jammers

Some parameters which are especially applicable to jammers for simulating different types of jammers are as given in Table 4.

**Figure 5.**
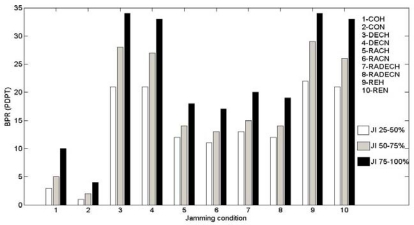
It shows the average Bad Packets Ratio (BPR) or Packets Dropped per Terminal (PDPT) for various jammers and jamming indices for simulation and sampling duration of 20 seconds.

The packets dropped per terminal (PDPT) for various types of jammers are as given in Figure 5.

The expansions of acronyms used in Figure 5 are: CON- Constant jammer with Normal Power, COH- Constant Jammer with high power, DECN- Deceptive Jammer with Normal Power, DECH-Deceptive Jammer with High Power, RACN- Random Jammer Imitating CON, RACH- Random Jammer Imitating COH, RADECN- Random Jammer Imitating DECN, RADECH- random Jammer Imitating DECH, REN- Reactive Jammer with Normal Power, and REH- Reactive Jammer with High Power.

### 6.3. Description

**Figure 6.**
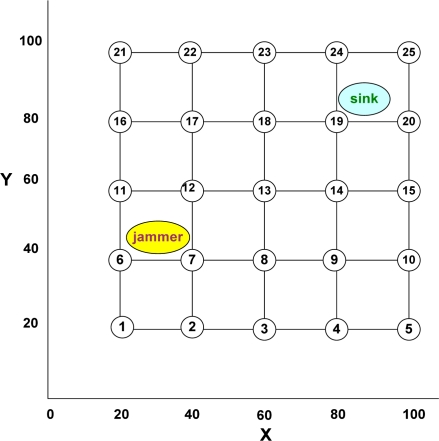
Schematic diagram of one of the 720 simulation set-ups, where the inter-nodal distance is 20 m, the jammer is a constant jammer with high output power, the jammer is located inside the WSN grid at coordinates (30,45) with the sink node located at coordinates (85,85), and the WSN has a total of 25 nodes, excluding the sink.

The grid topology for the WSN geographical extent was chosen to facilitate analysis of actual and predicted results. Six sets of inter-nodal distances: 5, 10,15,20,25, and 30 meters; and four positions for the jammer: two inside and two outside the grid were selected for the simulation. Three sets of total number (quantity) of nodes: 25, 50, and 100 were considered. Thus a total of 720 simulations (6 inter-nodal distances X 10 types of jammers X 4 jammer locations X 3 types of node quantity), with corresponding aforesaid parameters, were done using the NS2, MATLAB, and Simulink simulator. Figure 6 shows the schematic diagram of one of the 720 simulation set-ups, where the inter-nodal distance is 20m, the jammer is a constant jammer with high output power, the jammer is located inside the WSN grid at coordinates (30,45) with the sink node located at coordinates (85,85), and the WSN has a total of 25 nodes.

## 7. Results and Performance Evaluation

The results of the system are highly encouraging. We discuss the effects of various parameters on the result one by one while keeping the other parameters unchanged.

### 7.1. Inter-nodal Distances

We varied the inter- nodal distance from 5 to 30 meters and observed that the jamming indices of the nodes increased as we increased the inter-nodal distance and decreased when we decreased the inter-nodal distance. For example, the JIs in respect of Node-13 in the set-up described in Figure 6 were found to be 44.49, 62.93, 77.21, 88.95, and 98.27 for the inter-nodal distances of 5, 10, 15, 20, 25, and 30 m respectively, keeping the other factors unchanged. It indicates that a denser WSN is less vulnerable to jamming.

### 7.2. Jammer Type

We found that the effect of different type of jammers is different on the WSN and conforms to the expected pattern, wherein their effect, in order of decreasing intensity, is: REH, DECH, REN, DECN, RADECH, RADECN, COH, RACH, CON, and RACN. For example, the JIs in respect of Node-13 in the set-up described in Figure 6 were found to be 90.16, 90.15, 90.12, 90.11, 89.68, 89.14, 88.95, 88.72, 77.31, and 75.67 for REH, DECH, REN, DECN, RADECH, RADECN, COH, RACH, CON, and RACN respectively.

### 7.3. Jammer Location

We chose two locations inside and two locations outside the WSN extent randomly. We found that the jamming indices of nodes decreased when the jammer was farther and increased when the jammer was closer to the nodes. For example, the JIs in respect of Node-13 in the set-up described in Figure 6 were found to be 94.43, 88.95, and 77.62 for the node-to-jammer distances of 20, 30, and 40 m respectively, keeping the other factors unchanged.

### 7.4. Number of Nodes in the WSN

**Figure 7.**
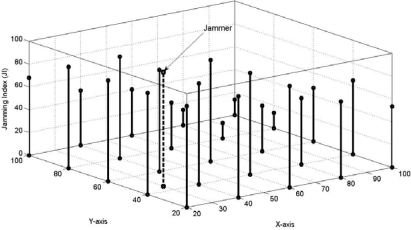
It shows the MATLAB simulation output for the set-up shown by Figure 6.

**Figure 8.**
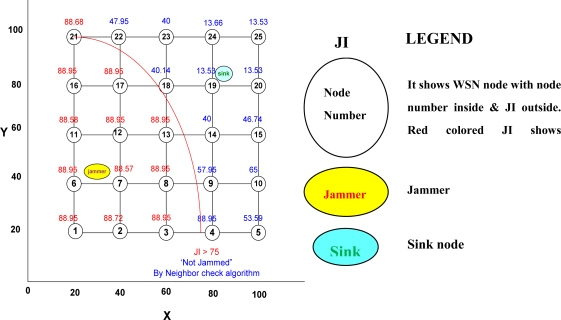
It graphically represents the output of the simulation for the set-up depicted by Figure 6.

We did not find any significant relation between the increase or decrease of jamming indices with increase or decrease of the number of nodes. However, we found that the number of jammed nodes increased with increase of the total number of nodes and it decreased as the latter was decreased, as long as the nodes were within the range of the jammer (40 m and 727 m for the low and high power jammers respectively). We now present the results. The MATLAB output of the simulation set-up, discussed in Figure 6, is given in Figure 7. Table 5 gives the output values of the simulation results of the set-up depicted in Figure 6 and Figure 8 represents the same graphically.

We find that there is a relationship between the various types of jammers with respect to the jamming indices, the power received at the nodes, and the inter-nodal distances in the WSN. We observe that it is possible to detect the type of jammers also through our method by using this relationship. The relationship is presented in Table 6. The relationship has to be read row-wise, e.g., the first row in the table will read, ‘If the PDPT of the node is from 5 to 10 AND the total received power by the node is 300 to 400 nW AND the inter-nodal distances of the node from its neighbors are 10 to 30 m, then the node is under the attack of a Constant Jammer.’ This table is relevant for all of the 720 simulations set-ups which we have described. Similar templates will have to be made for other set-ups. We find that our jammer discrimination results given in our Table 5 matches well with those of Xu *et al.* [1] given in their Figure 7.

### 7.5. Performance Evaluation

Performance evaluation of any model that detects a jamming attack is a difficult proposition because there is no known theoretical or practical model that can be taken as a bench-mark for comparison. It is perhaps because of this that all of the authors related with jamming detection quoted so far, except Cakiroglu *et al.* [3], have chosen not to evaluate their methods. Even the performance evaluation method described by Cakiroglu *et al.*, is ambiguous because they have neither defined the ‘jammed nodes ratio’ (ratio of the number of nodes successfully jammed by the jammer to the number of nodes covered by the jammer?) nor have they described the method to calculate it; but have used it to study the performance parameters, ‘detection rate’ and ‘false positive rate’, as a function of the ‘jammed nodes ratio’. We, therefore first define some terms which we use in our performance evaluation and then describe how these are calculated or obtained, where necessary. We then compare our results with those of Cakiroglu *et al.* [3].

*Jammed Nodes Ratio (jnr)* is mathematically defined as:

(10) $$\mathrm{jnr}=\frac{\mathrm{Number}\;\mathrm{of}\;\mathrm{nodes}\;\mathrm{successfully}\;\mathrm{jammed}\;\mathrm{by}\;\mathrm{the}\;\mathrm{jammer}}{\mathrm{Number}\;\mathrm{of}\;\mathrm{nodes}\;\text{cov}\mathrm{ered}\;\mathrm{by}\;\mathrm{the}\;\mathrm{jammer}\;\text{or}\;\mathrm{falling}\;\mathrm{within}\;\;\mathrm{the}\;\mathrm{range}\;\mathrm{of}\;\mathrm{the}\;\mathrm{jammer}}\cdot 100$$

where, ‘number of nodes successfully jammed by the jammer’ is the number of nodes which have been jammed as opined by the panel of experts on the basis of their jamming indices, the lower cut-off value of the jamming index as decided by the base station, and other aforesaid simulation parameters. The ‘number of nodes covered by the jammer’ is the number of nodes which fall within the communication range of the jammer (40 m and 727 m for the low and high power jammers respectively in our case).

*True Detection Ratio (TDR)* is defined as the ratio of the number of nodes correctly identified by the system to be falling under a jamming class (NORMAL, LOW, MEDIUM, or HIGH) to the number of such nodes as identified by the panel of experts, taken out of one hundred.

*False Detection Ratio (FDR)* is defined as the ratio of the number of nodes incorrectly identified by the system to be falling under a jamming class (NORMAL, LOW, MEDIUM, or HIGH) to the number of nodes actually falling under that group as identified by the panel of experts, taken out of one hundred.

We simulated 720 jamming scenarios, as described before. Each of these scenarios was also scrutinized by a panel of experts and their findings were obtained. The results as obtained from the system (simulation) and the experts were compared using statistical software, SPSS 11.5. We used the chi-square test for grouped comparison of data with degree of freedom (df) being 3 (as the number of groups are 4: NORMAL, LOW, MEDIUM, and HIGH), and level of significance (p) being 0.05 with the corresponding table value to be 7.815 giving a confidence interval of 95%. The test results for one of the simulations, as described in Figure 6 and Figure 8, are given in Table 7.

We mention here that 712 simulations (238 out of 240 for 25-node, 237 out of 240 for 50-node, and 237 out of 240 for100-node configurations) out of 720 simulations passed the chi-square test. The mean TDR and FDR from these 712 simulations were collated for different jammed node ratios (jnr) under different configurations (total number of nodes in the simulation, n) for different types of jammers. The values of TDR and FDR for 100 nodes configuration for different types of jammers for different types of jamming indices are given in Table 8 and Table 9, respectively.

**Figure 9.**
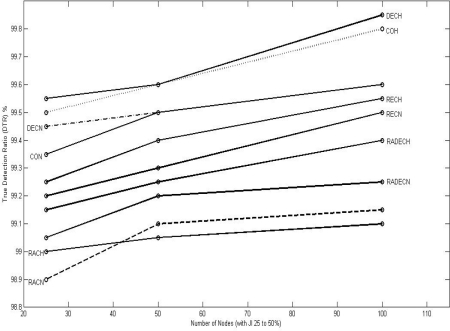
Graphic representation of TDR for different jnr for 100 nodes configuration for 25< JI < = 50.

**Figure 10.**
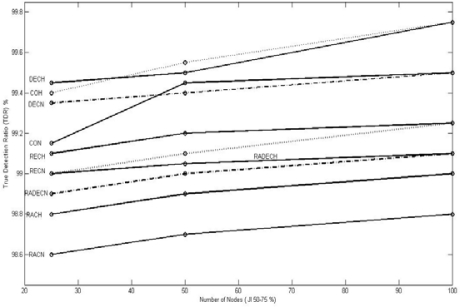
Graphic representation of TDR for different jnr for 100 nodes configuration for 50 < JI < = 75.

**Figure 11.**
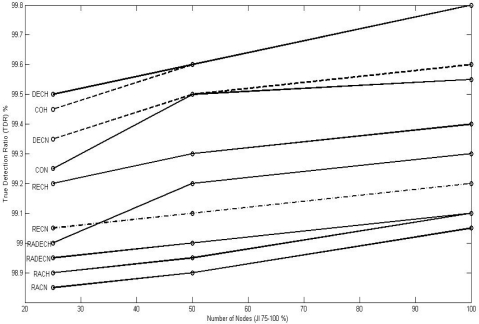
Graphic representation of TDR for different jnr for 100 nodes configuration for 75 < JI < = 100.

Figures 9, 10, and 11 show the values of TDR for 100 nodes configuration for different types of jammers and jnr for jamming indices of 25–50, 50–75, and 75–100% respectively.

We now compare our performance evaluation parameters, TDR and FDR with their corresponding counter-parts in the model by Cakiroglu *et al.*, ‘Detection Rate’ and ‘False Positive Rate’ in Tables 10 and 11 respectively, as they have now been reduced to an almost matching denominator. However, the comparison is to be studied with caution as the model by Cakiroglu *et al.* have preferred to use the Gilbert-Elliot model for simulating transmission losses based on two event discreet Markov chain, as they consider that the radio unit provides either good or bad transmission service.

We thus find that our performance parameters indicate good results and are either better or matching with the existing methods of jamming detection in wireless sensor networks whose figures are publically available.

We evaluated our approach with CBR traffic (a rather high traffic), and one may wonder whether the approach would also work for other traffic patterns, e.g., in a setting where the nodes usually do not communicate at all, or communicate only very rarely. In such a scenario, the PDPT could be very low, and in the worst case it could be zero. However, the SNR may or may not be low. Therefore, both PDPT and SNR will definitely have some value as defined by Equation 2 and Table 2. Accordingly, one of the nine rules given in the rule base under Section 5.1.1.2 (most probably, either of Rule 1, or Rule 4, or Rule 7) will be invoked, and the JI will be computed accordingly without any prejudice to the accuracy of the jamming detection. We thus conclude that the method is effective for all types of traffic patterns.

### 7.6. Evaluation of Base Station-Centric (Centralized) Versus Node-Centric (Decentralized) Approaches

We will now examine the performance of our proposed base-centric (centralized) approach vis-à-vis the existing approaches, all of them being node-centric (decentralized) from various angles as follows.

#### 7.6.1. Communication Energy Efficiency

We assume that the inter-nodal distance (hop distance) between any two nodes is the same.

Let:

*e* be the energy in joules required for transmission of one packet over one hop, and
*h* be the number of hops between a typical node to the base station.

In the proposed system, the node transmits only one additional packet containing its RSS value to the base station. If the nodes communicate frequently with the base station (sink) in the normal traffic pattern, the jamming-related data (the RSS packet) can be piggybacked with this traffic reducing the overhead. If, however, the normal traffic is very low, then the packet has to be sent independently which will increase the overhead. We will consider the latter case (the worse case). Let *f* be the number of such jamming-related data (the RSS packet) being sent by a typical node to the base station (sink) per second. Therefore, the total energy consumed per second for communication in the proposed centralized system, *Ec-cent* in joules is:

(11) Ec-cent=efh

Under the existing approaches (the decentralized approaches), e.g., in the models suggested by Xu *et al.* [1], Rajani *et al.* [2], and Cakiroglu *et al.* [3], the nodes have to communicate with their neighbors for neighborhood check or for sampling and threshold fixing. In these approaches, if a node has *n* neighbors, it has to send minimum one packet to each neighbor and receive one packet from each neighbor for neighborhood check. Let the frequency of this neighborhood-check (or, the number of time-windows during which metric-samples from neighbors are to be collected per second, as done in some cases, be *t* (*t* may be less than 1). Therefore, the minimum number of packets exchanged per hop per suspected jamming attack is *2nt.* If *j* is the average number of suspected jamming attacks per second, then the energy required for communication with the neighbors is *2ntej* joules per second. The nodes under the decentralized approach are then required to communicate to the base station if they detect jamming. Assuming that a fraction *k* of the *j* suspected jamming attacks are detected to be actual jamming, the node has to communicate *kj* times per second with the base station. Assuming further that the node sends only one packet per such communication, the energy required for this communication is *ejkh* joules per second. We thus have the total energy required for communication under the decentralized system in joules per second, *Ec-decent* given as:

(12) Ec-decent=2ntej+ejkh

For our proposed (centralized) system to be more communication energy efficient than the existing (decentralized) approaches, *Ec-cent* must be less than *Ec-decent*, *i.e.*,

(13) efh<2ntej+ejkh

(14) or,f<j(2nt/h+k)

Therefore, as evident from Inequality 14, our method (the centralized approach) can be only conditionally more communication energy-efficient than the existing (decentralized) approaches. It can thus be concluded that whether a distributed or centralized approach is more communication energy efficient depends on the communication pattern of the application.

#### 7.6.2. Computational Energy Efficiency

The energy consumption for computation of jamming detection for both approaches, centralized or decentralized, is the same for the same algorithm. However, the centralized system has the advantage of spending that energy from the base station and saving the same at the energy-starved nodes. Therefore, the centralized approach is decidedly better than the decentralized approach in this aspect.

#### 7.6.3. Speed of Jamming Detection

The decentralized approach is undoubtedly much faster than the centralized approach as the latter is based on distributed processing. However, the jamming detection is done for a greater purpose like mapping the jammed area, localizing and tracking the jammer, and taking counter-jamming actions at different layers. An over-all global picture must be built to meet the purpose. In the decentralized approach, the base station has to build-up the over-all picture almost de-novo, whereas, in the centralized approach, the over-all picture is almost simultaneously built along with the process of detection. This aspect compensates the slow speed of the centralized approach and places it at par with the decentralized approach.

Let us also do the time complexity analysis of our proposed method which uses two different algorithms: Fuzzy Inference System (FIS) and 2-Means Clustering (TMC).

For the FIS, the time function is given as:

(15) $$T(N)=k_{1}{\mathrm{Ns}}^{u}+k_{2},$$

where *N* is the number of iterations (*i.e.*, *N* is the number of nodes), *s* is the number of fuzzy sets defined for the input (*s* = 3 in our case, *i.e.*, LOW, MEDIUM, and HIGH), *u* is the number of inputs (*u* = 2 in our case, *i.e.*, SNR and PDPT), and *k_1_* and *k_2_* are constants. Therefore,

(16) $$T(n)=9k_{1}N+k_{2},$$

Therefore, the time complexity of FIS is of O(*N*).

In the TMC, we have four un-nested loops defined by equations 4 to 6, each running from 1 to *nj* or *(n–nj)*, where *n* and *nj* are the ‘number of one-hop neighbors’ and the ‘number of jammed one-hop neighbors’ respectively of the node under consideration. Since, the maximum value which *nj* or *(n–nj*) can take in these loops is bounded by *n/2*; the maximum number of possible iterations in any of these un-nested loops is limited to *n/2*. There are a total of *t* jammed nodes for which the TMC has to be applied. Therefore, the time taken by the algorithm, *T* is:

(17) $$T=k_{3}\;(n/2)\;t+k_{4},$$

where *k_3_* and *k_4_* are constants. Since both, t and n are bounded by the upper limit N, the time complexity of TMC is of O(*N^2^*). Thus, the overall time complexity of the two algorithms taken together is of O(*N^2^*).

In absolute terms, the total time taken to process 25 nodes in a WSN configuration as depicted in Figure 6 using vectors and ‘tic’ and ‘toc’ commands of MATLAB7 on a 2.4 GHz Pentium computer for FIS and TMC together was found to be 0.797 seconds.

#### 7.6.4. Accuracy of Detection

The centralized approach is decidedly more accurate than the decentralized approach because the former is based on local as well as global conditions while the latter is based only on the local conditions. This has amply been established by the high TDR and low FDR of our method, as discussed in preceding sections.

## 8. Conclusions and Future Work

We first discussed and analyzed the various types of jamming attack models, the determinant metrics for jamming detection, and the existing methods of jamming detection as applied to the wireless sensor networks. We also discussed how our approach to the problem differed from the existing ones from three angles: (1) the scope of the lethality of the jammer being enlarged to include military jammers, (2) the existing approaches consider only two discreet levels of jamming, jammed and not jammed; where as we consider that decision about whether a node is jammed or not jammed is a fuzzy one and accordingly, aim to grade different node as per their jamming indices which suits the information war environment allowing various options to the war- zone commander (or, base station) in adopting different policies with respect to victimized nodes, and (3) the decision for jamming detection is taken by the nodes themselves in the existing methods, which we consider not feasible due to the resource constraints of the WSN nodes and their ineffectiveness in communicating with other nodes during jamming, and accordingly, we choose to do all processing and decision making at the base station on a holistic picture. Having done so, we then selected packets dropped per terminal (PDPT) and signal-to-noise ratio (SNR) as the input to our fuzzy inference system based on Mamdani model which gave the jamming index (JI) of various nodes as output. This output is further evaluated on an overall picture based on the neighborhood of the nodes. We then evaluated the system performance through the true detection ratio (TDR) and false detection ratio (FDR), and then we compared the performance with the method proposed by Cakiroglu *et al.* and found that our performance is better in most of the cases, matching in few cases, and not as good as the others in rare cases. We are unable to compare our performance with those of other existing models because their quantitative performance evaluation parameters are not publically available. We also analyzed the performance of the proposed approach (centralized or base-centric approach) against the existing approaches (decentralized or node-centric approach) and found that the proposed approach is matching with the existing approaches in terms of speed and energy consumption and is better in terms of accuracy. Our model has the unique advantages of providing flexibility to the battle field commander (base station) in resource (node) utilization through grading the nodes with jamming indices, and of survivality in an information war, as the model is procedure-based as against protocol-based, with the latter involving inter-node communication in a jamming environment which may not be always possible. The proposed method has the additional advantage of being able to discriminate various types of jamming attacks without getting into the complexities of digital signal processing which must be avoided as they are not practicable in the WSN scenario in an information warfare environment.

We find that our algorithm for confirmation of jamming attack on a node through ‘2-means clustering’ of node neighborhood can be refined for its performance with respect to the edge nodes, especially those in the corners. We understand that this can be done by addition of steps for discriminating edge and corner nodes from the rest and allotting various allowances to them for loss of prospective jammed or un-jammed neighbors in our algorithm. We plan to undertake this task as our future work.

## Figures and Tables

**Table 1.** A comparative study of existing jamming detection methods.

**Comparison parameter**	**Xu*et al.***	**Rajani*et al.***	**Cakiroglu*et al.***	**Reese*et al.***	**Strasser*et al.***
Jamming detection done by	Individual nodes	Individual nodes	Individual nodes	Individual nodes	Individual nodes
Requirement for sampling and threshold fixing	Not required	Required	Required	Required	Required
Requirement for neighborhood check or threshold fixing	Required	Required	Required	Required	Required
Requirement to communicate with neighbors during jamming	Required	Required	Required	Required	Required
Requirement to communicate with base-station to report jamming	Required	Required	Required	Required	Required
Ability to discriminate different types of jamming	Able	Unable	Unable	Unable	Unable
Node over-load assessment (1: minimum, 5: maximum)	2	5	1	3	4
Accuracy assessment (1: most accurate, 5: least accurate)	3	5	2	4	1
Speed assessment (1: fastest, 5: slowest)	3	5	4	1	2

**Table 2.** Values of variables used in definition of membership functions.

**Universe of discourse (uod)**	**Set**	**a**	**b**	**c**	**d**
SNR	LOW	−0.5	0	1	1.5
	MEDIUM	1	1.5	10	12
	HIGH	10	12	3,900	4,000
PDPT	LOW	−5	0	10	15
	MEDIUM	10	15	25	30
	HIGH	25	30	50	55
JI	NO	−5	0	25	30
	LOW	25	30	50	55
	MEDIUM	50	55	75	80
	HIGH	75	80	100	105

**Table 3.** Parameters used for simulating WSN and those parameters which are used for simulating jammers.

**Parameter**	**WSN**	**Jammer**

Frequency (f)	914.634 MHz	914.634 MHz
Wavelength (λ)	0.328 m	0.328 m
Antenna gain	1 (0dB)	1 (0dB)
Antenna directivity	Omni directional	Omni directional
Transmitted power (Pt)	8.56 × 10^−4^ W	Variable
Receiver sensitivity (Prth)	3.652 × 10^−4^ W	3.652 × 10^−4^ W
Maximum radio range	40 m	40 m
Propagation model	Free space	Free space
Path loss (L)	1 (0dB)	1 (0dB)
Mode of transmission	Simplex unicast	Simplex broadcast
Packet size	1000 B	Variable
Transmission rate	0.01 MBPS	Variable
Application layer protocol	CBT	CBT
Transport layer protocol	UDP	UDP
Network layer protocol (routing Protocol)	AODV	AODV
MAC protocol	BMAC.	BMAC

**Table 4.** Parameter values for simulating different types of jammers.

**Type of jammer**	**Output Power (W)**	**Packet Size (MB)**	**Rate (MBPS)**	**Transmission Duration**
Constant Jammer with Normal Power (CON)	8.56 × 10^−4^	10,000	10	constant
Constant Jammer with High Power (COH)	0.2818	10,000	10	Constant
Deceptive Jammer with Normal Power (DECN)	8.56 × 10^−4^	1,000	0.01	Constant
Deceptive Jammer with High Power (DECH)	0.2818	1,000	0.01	Constant
Random Jammer Imitating CON, (RACN)	8.56 × 10^−4^	10,000	10	Random
Random Jammer Imitating COH, (RACH)	0.2818	10,000	10	Random
Random Jammer Imitating DECN, (RADECN)	8.56 × 10^−4^	1,000	0.01	Random
Random Jammer Imitating DECH, (RADECH)	0.2818	1,000	0.01	Random
Reactive Jammer with Normal Power (REN)	8.56 × 10^−4^	1,000	0.01	Whenever there is a legitimate transmission between any source and the sink.
Reactive Jammer with High Power (REH)	0.2818	1,000	0.01	Whenever there is a legitimate transmission between any source and the sink.

**Table 5.** Output values of the simulation for the set-up depicted in Figure 6.

**Node No.**	**X-coord**	**Y-coord**	**Power received w/o jammer *Pnj (nW)***	**Power received with jammer *Pj (nW)***	**Power received due to jammer *Prdj=(Pj- Pnj) (nW)***	**Signal-to-noise ratio *SNR***	**Packets dropped per terminal *PDPT***	**Jammin g index *JI***	**Decision for *LC=75* w/o neighborhood check**	**Decision with neighborhood check for LC=75**

1	20	20	29.45	294.254	264.808	0.111	9.401	88.95	Jammed	Jammed
2	40	20	39.80	304.609	264.808	0.150	10.998	88.72	jammed	jammed
3	60	20	49.13	175.024	125.892	0.387	20.606	88.95	jammed	jammed
4	80	20	54.19	115.692	61.435	0.869	17.518	88.95	Jammed	Not jammed
5	100	20	49.87	84.617	34.749	1.394	11.429	53.59	Not jammed	Not jammed
6	20	40	39.71	1575.69	1535.886	0.026	15.584	88.95	Jammed	Jammed
7	40	40	59.46	1595.35	1535.886	0.039	11.685	88.57	Jammed	Jammed
8	60	40	85.08	292.634	207.552	0.408	9.298	88.95	Jammed	Jammed
9	80	40	105.7	181.73	76.034	1.372	12.257	57.95	Not jammed	Not jammed
10	100	40	94.41	133.392	38.982	2.361	21.084	65	Not jammed	Not jammed
11	20	60	49.13	639.858	590.725	0.083	11.655	88.58	Jammed	Jammed
12	40	60	85.09	675.813	590.725	0.144	16.334	88.95	Jammed	Jammed
13	60	60	206.9	377.517	170.654	0.972	7.818	88.95	Jammed	Jammed
14	80	60	308.0	378.456	70.453	4.310	7.576	40	Not jammed	Not jammed
15	100	60	235.4	272.874	37.461	6.120	11.307	46.74	Not jammed	Not jammed
16	20	80	54.30	199.151	144.895	0.372	21.363	88.95	Jammed	Jammed
17	40	80	105.7	250.591	144.895	0.724	15.084	88.95	Jammed	Jammed
18	60	80	308.0	398.349	90.346	3.372	10.027	40.14	Not jammed	Not jammed
19	80	80	3852	3903.30	51.534	73.31	6.160	13.53	Not jammed	Not jammed
20	100	80	777.0	808.371	31.345	24.02	6.821	13.53	Not jammed	Not jammed
21	20	100	49.87	111.304	61.435	0.799	24.425	88.95	Jammed	Jammed
22	40	100	94.41	155.845	61.435	1.512	11.553	47.95	Not jammed	Not jammed
23	60	100	235.4	284.327	48.914	4.716	8.515	40	Not jammed	Not jammed
24	80	100	777.0	811.775	34.749	21.74	10.025	13.66	Not jammed	Not jammed
25	100	100	433.4	457.586	24.225	17.18	8.913	13.53	Not jammed	Not jammed

**Table 6.** Inter-parameter relationships for detecting the type of jamming attack.

**PDPT**	**Total power received (nW)**	**Inter-nodal distance (m)**	**JI**	**Type of jamming/condition**

5 to10 AND	300 to 400 AND	10 to 30 AND	75 to 90	Constant
> 20 AND	10 to 300 AND	5 to 30 AND	90 to 100	Deceptive
> 20 AND	> 400 AND	5 to 10 AND	90 to 100	Reactive
10 to 20 AND	> 400 AND	5 to 10 AND	50 to 75	Random
0 to10 AND	10 to 4000 AND	5 to 30 AND	< =50	Normal condition

**Table 7.** Test results for one of the simulations, as described in Figure 6 and Figure 8. The results pass the chi-square test as the total under (O-E)^2^/E, 0 is less than the table value of 7.815.

**Group of JI**	**No. of nodes placed by system (O)**	**No. of nodes placed by experts (E)**	**(O-E)^2^/E**	**No. of nodes correctly placed by system (T)**	**No. of nodes incorrectly placed by system (F)**	**TDR = 100.T/E**	**FDR = 100.F/E**	**Jnr = 100.E/25**

Normal	4	4	0	4	0	100%	0%	16%
Low	5	5	0	5	0	100%	0%	20%
Medium	4	4	0	4	0	100%	0%	16%
High	12	12	0	12	0	100%	0%	48%
Total	25	25	0	25	0	-	-	-

**Table 8.** It shows the values of TDR for 100 nodes configuration for different types of jammers, JIs and jnr.

**Jammer Type**	**True Detection Ratio (TDR %) for 100 nodes configuration**								

	**25 < JI < =50**			**50 < JI < = 75**			**75 < JI < = 100**		

	**jnr 25%**	**jnr 50%**	**jnr 100%**	**jnr 25%**	**jnr 50%**	**jnr 100%**	**jnr 25%**	**jnr 50%**	**jnr 100%**

DECH	99.55	99.6	99.85	99.45	99.5	99.75	99.50	99.6	99.8
COH	99.5	99.6	99.8	99.40	99.55	99.75	99.45	99.6	99.8
DECN	99.45	99.5	99.6	99.35	99.4	99.5	99.35	99.5	99.6
CON	99.35	99.5	99.6	99.15	99.45	99.5	99.25	99.5	99.55
REH	99.25	99.4	99.55	99.10	99.2	99.25	99.20	99.3	99.4
REN	99.20	99.3	99.5	99.00	99.1	99.25	99	99.2	99.3

RADECH	99.15	99.25	99.4	99.00	99.05	99.10	99.05	99.1	99.2
RADECN	99.05	99.20	99.25	98.90	99	99.10	98.95	99	99.1
RACH	98.90	99.10	99.15	98.80	98.90	99	98.90	98.95	99.1
RACN	99.00	99.05	99.10	98.60	98.70	98.8	98.85	98.9	99

**Table 9.** It shows the values of FDR for 100 nodes configuration for different types of jammers, jnr and JIs.

**Jammer Type**	**False Detection Ratio (FDR %) for 100 nodes configuration**								

	**25 < JI < = 50**			**50 < JI < = 75**			**75 < JI < = 100**		

	**jnr 25%**	**jnr 50%**	**jnr 100%**	**jnr 25%**	**jnr 50%**	**jnr 100%**	**jnr 25%**	**jnr 50%**	**jnr 100%**

RADECH	0.6	0.3	0	0.7	0.4	0	0.55	0.25	0
RADECN	0.5	0.25	0	0.6	0.3	0	0.5	0.2	0
DECH	0.45	0.2	0	0.5	0.3	0	0.4	0.1	0
COH	0.3	0.01	0	0.35	0.02	0	0.2	0	0
REH	0.25	0.1	0	0.28	0.12	0	0.2	0.1	0
RACH	0.05	0.04	0	0.06	0.05	0	0.04	0.03	0
RACN	0.03	0.02	0	0.04	0.03	0	0.03	0.01	0
REN	0.01	0.01	0	0.02	0.02	0	0.01	0.01	0
DECN	0.01	0.01	0	0.02	0.01	0	0.01	0	0
CON	0.01	0.01	0	0.01	0.01	0	0.01	0	0
DECH	0.45	0.2	0	0.5	0.3	0	0.4	0.1	0
DECN	0.01	0.01	0	0.02	0.01	0	0.01	0	0

**Table 10.** Comparison of TDR% (proposed model) and ‘Detection Rate %’(model by Cakiroglu *et al.*). The asterisk mark (*) shows those readings where the model by Cakiroglu *et al.* is better than the proposed model. Elsewhere, the proposed model’s performance is either matching or better.

**Type of jammer**		**TDR% (proposed model) and ‘Detection Rate %’(model by Cakiroglu *et al.*)**					

**Proposed model**	**Cakiroglu *et al.* equivalent**	**jnr 25%**		**jnr 50%**		**jnr 100%**	

		**Proposed**	**Cakiroglu**	**Proposed**	**Cakiroglu**	**Proposed**	**Cakiroglu**

DECH	Deceptive (bad)	99.45	99.35	99.5	99.38	99.75	99.44
COH	Constant (bad)	99.40	99.32	99.55	99.37	99.75	99.42
DECN	Deceptive	99.35	99.25	99.40	99.29	99.50	99.43
CON	Constant	99.15	99.20 *	99.45	99.28	99.50	99.34
REH	Reactive (bad)	99.10	99.15*	99.20	99.18	99.25	99.32*
REN	Reactive	99.00	99.05*	99.10	99.10	99.25	99.25
RADECH	Random (bad)	99.00	99.06*	99.05	99.06*	99.10	99.16*
RADECN	-	98.90	-	99.00	-	99.10	-
RACH	-	98.80	-	98.90	-	99.00	-
RACN	Random	98.60	98.82*	98.70	98.90*	98.80	99.10*

**Table 11.** Comparison of FDR% (proposed model) and ‘False Positive Rate %’(model by Cakiroglu *et al.*). The proposed model’s performance values are either better or matching with those of the model by Cakiroglu *et al.*

**Type of jammer**		**FDR% (proposed model) and ‘False Positive Rate %’(model by Cakiroglu *et al.*)**					

**Proposed model**	**Cakiroglu *et al.* equivalent**	**jnr 25%**		**jnr 50%**		**jnr 100%**	

		**Proposed**	**Cakiroglu**	**Proposed**	**Cakiroglu**	**Proposed**	**Cakiroglu**

RADECH	Random (bad)	0.7	0.8	0.4	0.51	0	0
RADECN	-	0.6	-	0.3	-	0	-
DECH	Deceptive (bad)	0.5	0.57	0.3	0.4	0	0
COH	Constant (bad)	0.35	0.38	0.02	0.04	0	0
REH	Reactive (bad)	0.28	0.3	0.12	0.13	0	0
RACH	-	0.06	-	0.05	-	0	-
RACN	Random	0.04	0.05	0.03	0.03	0	0
REN	Reactive	0.02	Not clear	0.02	Not clear	0	0
DECN	Deceptive	0.02	Not clear	0.01	Not clear	0	0
CON	Constant	0.01	Not clear	0.01	Not clear	0	0
